# Supplementary material for: Immunogenicity, Efficacy, and Safety of a Novel Synthetic Microparticle Pre-Erythrocytic Malaria Vaccine in Multiple Host Species
Source: Vaccines (Basel). 2023 Nov 30;11(12):1789. doi: 10.3390/vaccines11121789 (PMC10748200; doi:10.3390/vaccines11121789)
Supplement: Supplementary file 1 [file vaccines-11-01789-s001.zip › vaccines-2705946-Supplementary.pdf]

Supplementary Figure S1.

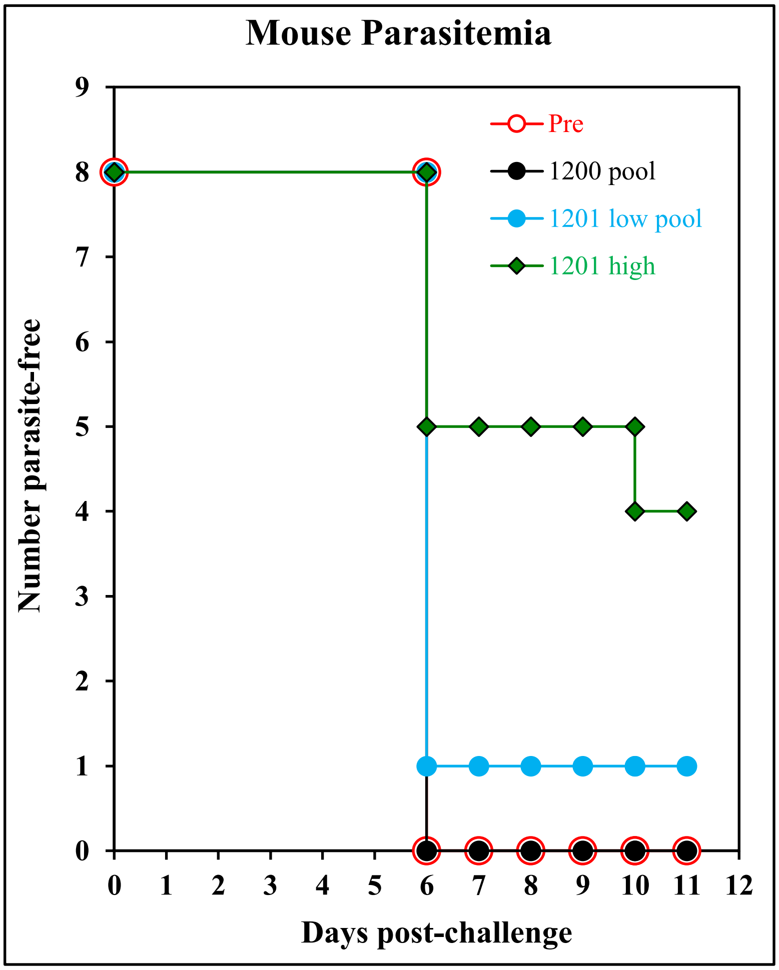

Supplemental Figure S1. Preliminary efficacy in NHPs. Macaques were immunized i.d. with 50 μg of ACT-1200 or ACT-1201 on days 0, 28, and 56 (as indicated in Figure 4), and bled on days 14, 42, and 70 (14 days following each immunization). Day 70 sera from each NHP were tested in ELISA (see Figure 4A, B) and pooled according to the antibody response. Pre = pool of pre-bleed sera from 8 NHP. 1200 pool = pool of all 4 sera from the ACT-1200-immunized NHP. 1201 low pool = pool of sera from the 3 lowest responders in the ACT-1201 group (animals 08U027, 08U029, and 09U044). 1201 high = sera from the ACT-1201 NHP that had the highest ELISA response (animal 08031). Ig was purified from each serum pool and administered to 8 naïve mice per donor pool prior to the challenge with live Tg-*Pb*/*Pf*CSP parasite, and parasitemia was monitored post-challenge. Results show the number of mice per group that remained parasite-free as determined by microscopic examination of Geimsa-stained blood smears.

Supplemental Figure S2.

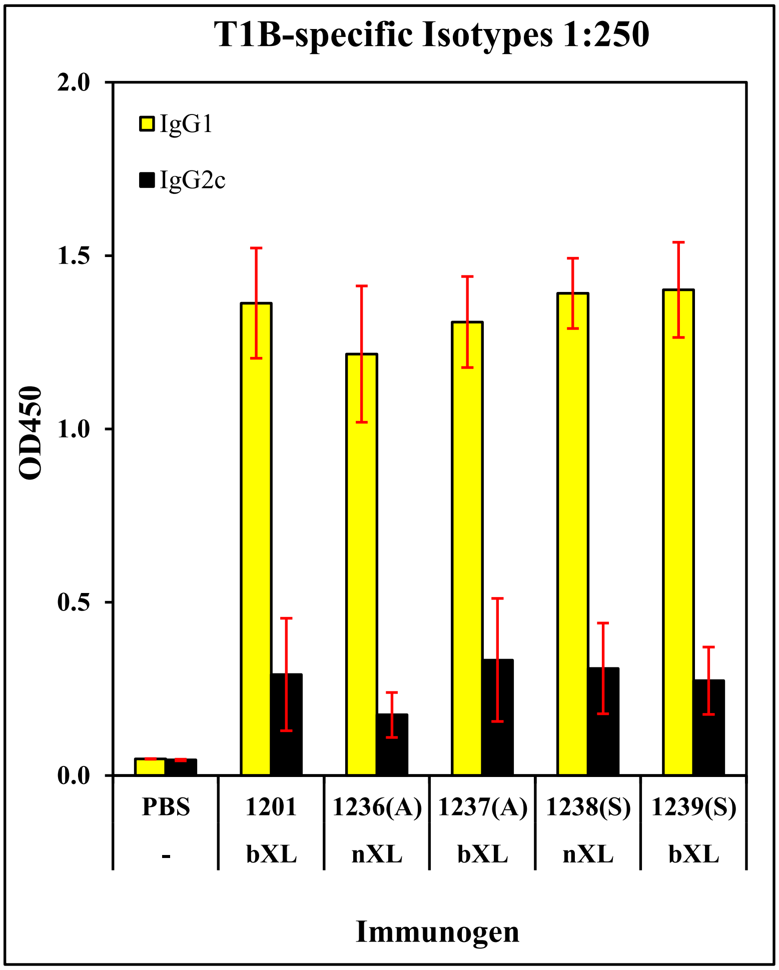

Supplemental Figure S2. Sera from Figure 6 were harvested on day 59 and tested in ELISA against T1B peptide at a serum dilution of 1:250. Plates were probed with isotype-specific detection antibodies. The data depict the mean ± SD of 10 mice per group.
